# Disrupting the MAD2L2-Rev1 Complex Enhances Cell Death upon DNA Damage

**DOI:** 10.3390/molecules27030636

**Published:** 2022-01-19

**Authors:** Nomi Pernicone, Maria Elias, Itay Onn, Dror Tobi, Tamar Listovsky

**Affiliations:** 1 Department of Molecular Biology, Ariel University, Ariel 40700, Israel; neomipe@ariel.ac.il; 2 Azrieli Faculty of Medicine, Bar Ilan University, Safed 52900, Israel; maria.elias.1007@gmail.com (M.E.); Itay.Onn@biu.ac.il (I.O.); 3 Department of Computer Science, Ariel University, Ariel 40700, Israel; 4 The Ariel Center for Applied Cancer Research (ACACR), Ariel University, Ariel 40700, Israel; 5 Adelson School of Medicine, Ariel University, Ariel 40700, Israel

**Keywords:** TLS, MAD2L2, small molecules

## Abstract

DNA-damaging chemotherapy agents such as cisplatin have been the first line of treatment for cancer for decades. While chemotherapy can be very effective, its long-term success is often reduced by intrinsic and acquired drug resistance, accompanied by chemotherapy-resistant secondary malignancies. Although the mechanisms causing drug resistance are quite distinct, they are directly connected to mutagenic translesion synthesis (TLS). The TLS pathway promotes DNA damage tolerance by supporting both replication opposite to a lesion and inaccurate single-strand gap filling. Interestingly, inhibiting TLS reduces both cisplatin resistance and secondary tumor formation. Therefore, TLS targeting is a promising strategy for improving chemotherapy. MAD2L2 (i.e., Rev7) is a central protein in TLS. It is an essential component of the TLS polymerase zeta (ζ), and it forms a regulatory complex with Rev1 polymerase. Here we present the discovery of two small molecules, c#2 and c#3, that directly bind both in vitro and in vivo to MAD2L2 and influence its activity. Both molecules sensitize lung cancer cell lines to cisplatin, disrupt the formation of the MAD2L2-Rev1 complex and increase DNA damage, hence underlining their potential as lead compounds for developing novel TLS inhibitors for improving chemotherapy treatments.

## 1. Introduction

DNA-damaging platinum-based chemotherapeutics (cisplatin, carboplatin, oxaliplatin) have been the first line of treatment for cancer for decades. They act by binding or modifying DNA and blocking replication, generating cytotoxicity and apoptosis of rapidly dividing cancer cells [1,2]. While chemotherapy can be very effective, its long-term success is often reduced by intrinsic and acquired drug resistance. Moreover, chemotherapy promotes the formation of treatment-based mutations that can lead to chemotherapy resistance of secondary malignancies, increasing morbidity in many cancer patients. Although the mechanisms causing intrinsic and acquired drug resistance are quite distinct, they are directly connected to mutagenic translesion synthesis (TLS). The TLS pathway is an essential cellular mechanism that allows DNA damage tolerance by supporting DNA replication and single-strand gap filling opposite to DNA lesions without repairing the damage [3,4,5,6]. Therefore, cells rely on an active TLS pathway to survive cisplatin cytotoxicity despite the cost of introducing new mutations. The TLS family contains error-prone specialized polymerases. Specifically, it includes Y-family polymerases such as Rev1, Pol eta (η), Pol iota (ι), Pol kappa (κ) and the B-family polymerase Pol zeta (ζ). TLS is divided into a two-step mechanism, in which usually a Y-family polymerase replicates through the lesion, followed by the extension of the distorted DNA structure by Pol ζ. The Pol ζ core complex contains two proteins, the polymerase catalytic subunit Rev3 and a regulatory subunit MAD2L2 (i.e., Rev7) that stabilizes the polymerase and facilitates its interaction with Rev1 [7,8,9,10]. TLS activity in vivo depends on MAD2L2 homodimerization and binding to Rev1 for recruitment of the proper Y-family polymerase [9,11]. Although the TLS activity of specific insertion DNA polymerases over their matching lesions can be relatively accurate [12], the major mutagenesis generated by Rev1 and Pol ζ activity can cause intrinsic and acquired drug resistance and reduce chemotherapy success. Overall, the TLS pathway allows cells to tolerate DNA damage, which has a net benefit, as mutagenic replication is preferable to fork collapse and incomplete replication, which may cause chromosomal instability [13]. Indeed, the loss of proteins involved in TLS can lead to diseases that exhibit DNA instability, such as Fanconi anemia or a variant of xeroderma pigmentosum (XP-V) [14,15]. Therefore, inhibiting TLS during chemotherapy treatment can suppress lesion bypass, increase DNA damage and accelerate cell death. Recent studies showed that genetic inhibition of TLS through RNA-mediated depletion of Rev1, Rev3 or MAD2L2 indeed sensitizes a variety of cancer cells to DNA-damaging chemotherapeutics and suppresses the emergence of new tumor chemoresistance both in cancer cell lines and animal models [16,17,18,19]. Identifying small molecules with an efficient in-vivo effect on TLS inhibition is a challenging task with very few successes. The JH-RE-06 molecule is an excellent example of a very potent molecule. It causes Rev1 dimerization, preventing MAD2L2-Rev1 binding and inhibiting TLS activity [16]. Here, we present two small molecules, c#2 and c#3, that were identified out of close to five million possible lead compounds and potentially cause TLS inhibition. We used high-throughput virtual screening against a model of the MAD2L2 homodimer generated by molecular dynamics simulation. We found that both molecules disrupt MAD2L2 activity by binding to MAD2L2 and preventing the MAD2L2-Rev1 complex formation. In addition, combined treatment of cisplatin and c#2 or c#3 sensitizes the cells and enhances DNA damage and cell death. Further development of these lead compounds offers a new therapeutic strategy as TLS inhibitors for the improvement of cancer therapy.

## 2. Results

### 2.1. MAD2L2-MAD2L2-Rev1 Model and Docking Simulations

**Figure 1:**
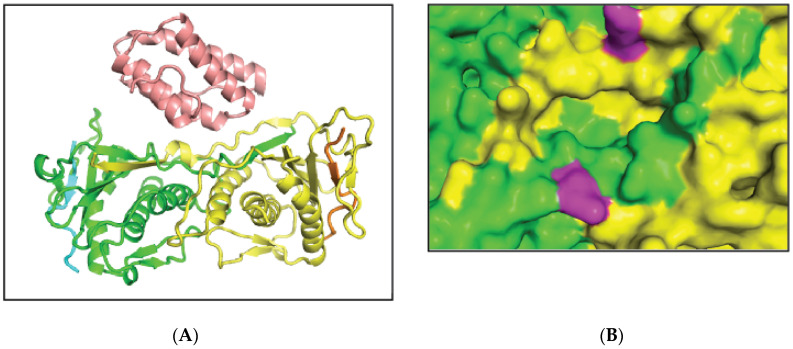
MAD2L2 dimer in complex with a CAMP fragment and Rev1. (**A**) MAD2L2 proteins are shown in cartoon representation: MAD2L2 (yellow and green), CAMP fragment (orange and cyan) and Rev1 (Pink). (**B**) Surface representation of the cavity between the MAD2L2 monomers after binding Rev1. MAD2L2 monomers are colored yellow and green, and K190 of each monomer is colored magenta.

The dimeric form of MAD2L2 in complex with a CAMP [20,21] fragment and Rev1 was built as described in Methods. The complex was placed in a box of water, and a 30 ns-long molecular dynamics simulation (MD) was conducted to determine the structural changes that occur upon binding of Rev1 to the MAD2L2 dimer. A representative conformation (see Methods) from the last 1ns was selected (Figure 1A). Most of the interactions between Rev1 and the MAD2L2 dimer are at residues L186-K198 of one of the MAD2L2 monomers and K198-Y202 of the other monomer. K190 is one of the residues important for MAD2L2 dimerization [22]. Thus, despite the 2-fold symmetry of the MAD2L2 dimer [21], Rev1 binds to it asymmetrically. During the MD simulation, the cavity between the two MAD2L2 proteins became slightly wider. Binding of Rev1 results in an induced fit of the MAD2L2 dimer. The Cα-Cα distance between K190 of the 2 monomers increases from 15.86 to 18.10 Å, and the sidechains undergo reorientation for optimal binding of MAD2L2 (Figure 1B).

**Figure 2:**
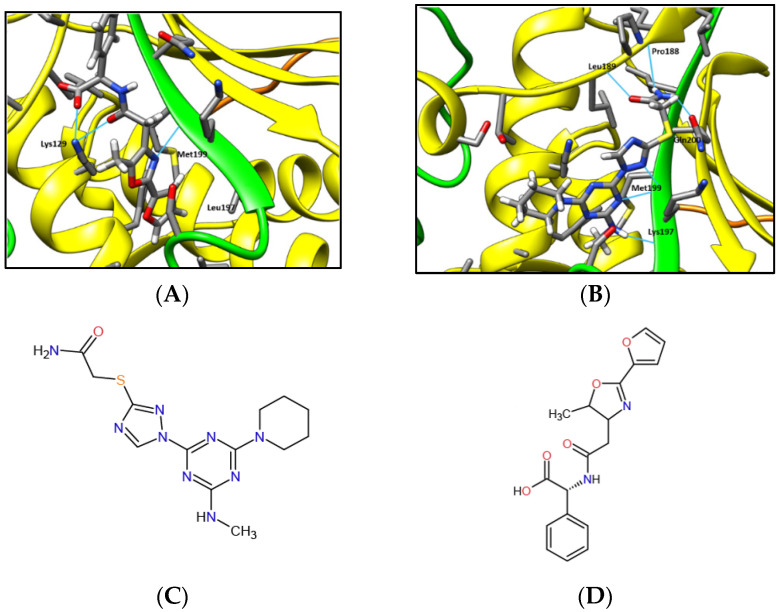
Small molecules docked in the cavity of the MAD2L2 dimer. The coloring scheme for MAD2L2 follows that of Figure 1. Molecules are shown using stick representation. (**A**) The small molecule ZINC97017995 (c#2) docked in the cavity of the MAD2L2 dimer. Met199 and Leu197 form Van der Waals or hydrophobic interactions with ZINC97017995. Lys129 forms two hydrogen bonds with the molecule. Met199 forms one hydrogen bond through its backbone. Hydrogen bonds are marked with a cyan line. (**B**) The small molecule ZINC25496030 (c#3) docked in the cavity of the MAD2L2 dimer. Met199 forms two hydrogen bonds with ZINC25496030. Pro188, Leu189, Lys197, and Gln200 each form one hydrogen bond with the molecule. (**C**) Two-dimensional drawing of ZINC97017995 (c#2). (**D**) Two-dimensional drawing of ZINC25496030 (c#3).

High-throughput virtual screening was conducted using DOCK6 software with the ZINC12 Clean Leads subset to identify potential lead compounds that bind to the reshaped cavity. Docking was done in two stages. Initially, all molecules were docked to the cavity using the fast anchor-and-grow algorithm with a rigid receptor and flexible ligand. The top 5000 ranked compounds were docked and rescored using AMBER force files with default scoring parameters allowing flexibility for the ligand and receptor amino acids contacting the ligand (see Methods). The best-ranking ligands were manually selected based on their ability to interfere with the interaction of Rev1 with the MAD2L2 dimer. Representative docking and biochemical interactions of two selected compounds, ZINC97017995 (c#2) (Figure 2A) and ZINC25496030 (c#3) (Figure 2B), in the MAD2L2 dimer cavity are presented. Two-dimensional drawings of c#2 and c#3 are presented in Figure 2C,D, respectively.

### 2.2. Both c#2 and c#3 Sensitize Cells to Cisplatin

**Figure 3:**
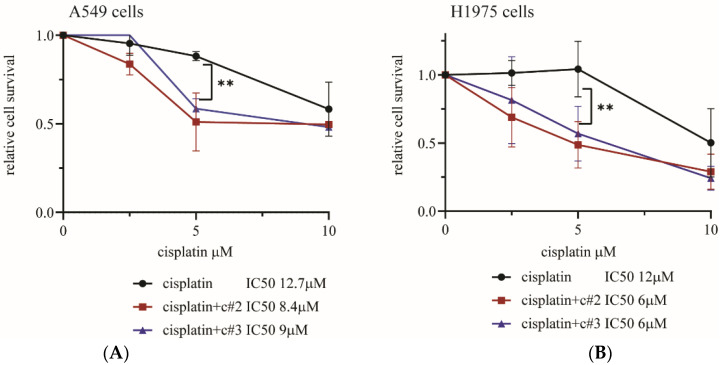
Compounds #2 and #3 sensitize cells to cisplatin treatment. (**A**) Colony survival assay of A549 cell line. (**B**) Colony survival assay of H1975 cell line. Both cell lines were treated with 50 μM of compound and the indicated concentration of cisplatin for 48 h; then, growth media was replaced with normal growth media with no compound. Cells were allowed to recover 4–5 days before staining. For both cell lines, colony survival assay *n* = 3 independent experiments, SD = 1, error bars represent 1 SD. *p*-value < 0.01 (**) was calculated by two-way ANOVA multiple comparisons. IC50 was calculated using linear regression. A representative colony survival assay is presented in Figure S1C.

In order to assess the ability of c#2 and c#3 to sensitize cells during cisplatin treatment, we established a colony survival assay to compare the relative survival rate of cells treated with cisplatin alone or with combined treatment of cisplatin and 50 μM c#2 and c#3. Survival was assessed in two lung cancer cell lines: human non-small cell lung carcinoma H1975 cells and epithelial lung adenocarcinoma A549 cells. Notably, treatment with c#2 or c#3 alone caused no toxicity during the colony survival assay (Figure S1A). However, when cells were exposed to a combined treatment of cisplatin and 50 μM of c#2 or c#3, an approximately twofold sensitization effect was observed. Interestingly, both cell lines presented close to 100% relative survival rate during 5 μM cisplatin treatment; however, their sensitivity was significantly reduced to 50% relative survival rate when exposed to combined treatment (Figure 3A,B). H1975 cells exhibit similar behavior following doxorubicin treatment (Figure S1B). The A549 cells presented partial cisplatin resistance and failed to completely die, even at high concentrations of cisplatin. Interestingly, the A549 cells also presented doxorubicin resistance (not shown). The relative enhanced cell death can also be observed by the reduction of cisplatin’s IC50 in all treatments. Notably, we focused on enhancing cell death while using low cisplatin concentrations, which normally have little effect on cell death. Lowering drug concentration during treatment has clear benefits for the patient in reducing side effects. To conclude, these results suggest that combined treatment of cisplatin with c#2 or c#3 is indeed effective in enhancing cell death at low cisplatin concentrations.

### 2.3. DNA Damage Is Elevated after Co-Treatment of Cisplatin Together with c#2 or c#3

**Figure 4:**
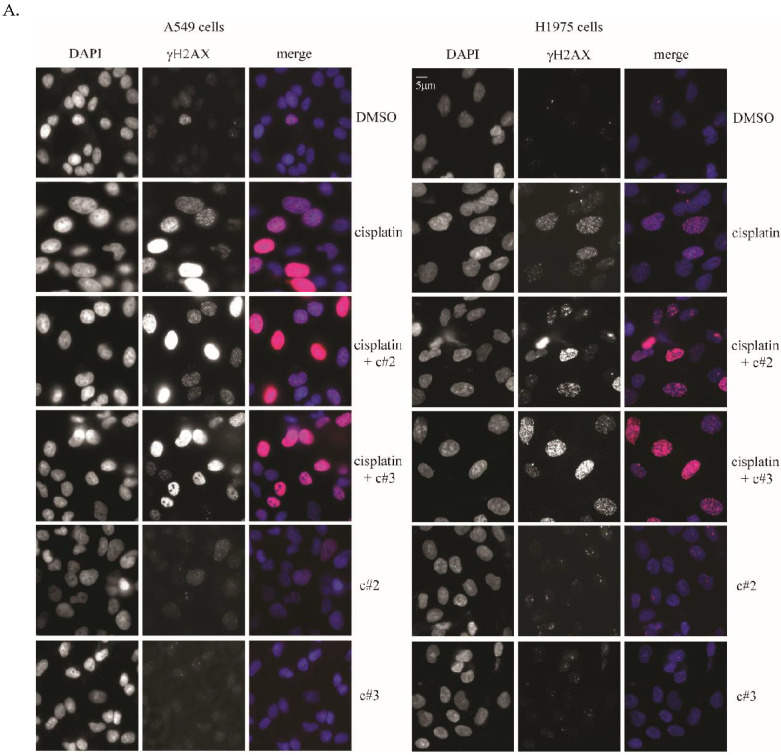

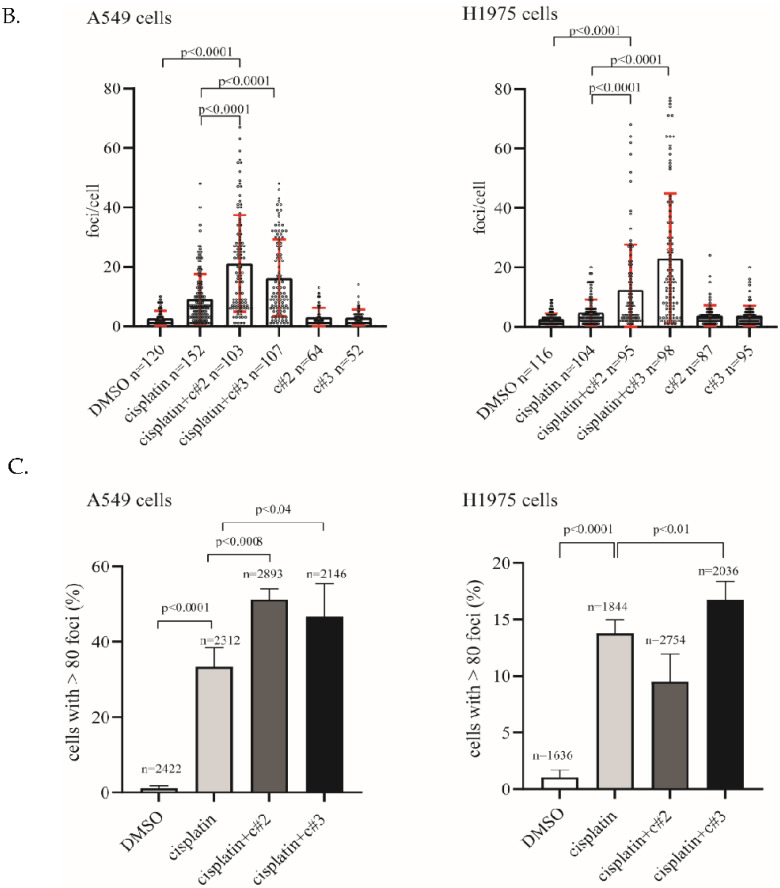
Compounds #2 and #3 increase DNA damage after combined treatment. (**A**) Representative figures of γH2AX in A549 (left panel) cells and H1975 (right panel) with different treatments as indicated. (**B**) Quantification of A549 and H1975 cells foci/cell in each treatment. The total number of cells scored is indicated. Error bars = 1 SD, error bars represent 1 SD. *p*-value was calculated by two-tailed *t*-test. (**C**) Quantification of A549 (left panel) and H1975 (right panel) cells with more than 80 foci/cell in each treatment. The total number of cells scored is indicated. These data were derived from four captures, in each of which at least 400 cells were scored. SD = 1, error bars represent 1 SD, for the independently determined percentages from the two experiments. *p*-value was calculated using a one-tailed *t*-test. A549 cells were treated with 10 μM cisplatin and H1975 cells with 5 μM cisplatin. Both cell lines were treated with 50 μM of c#2 or c#3.

Next, we examined whether combined cisplatin treatment with c#2 or c#3 correlates with an increase in DNA damage, which might explain the compound’s sensitization effect. Levels of γ-H2AX foci, a marker for DNA double-strand breaks, were compared in A549 and H1975 cell lines 16 h after treatment with cisplatin or combined treatment (Figure 4A; A549 left panel, H1975 right panel). Combined treatment presented a 2-fold increase in γ-H2AX foci/cell compared to cisplatin treatment only (Figure 4B; A549 left panel, H1975 right panel). Conversely, 16h treatment of c#2 or c#3 on their own had no effect on γ-H2AX signal, and no cell death was observed. Interestingly, we noticed that the combined treatment induced the appearance of cells with more than 75–80 foci or very bright signal. These cells were quantified using ImageJ after careful threshold settings and excluding cells that presented apoptotic bodies. Here, we were able to quantify about 2000 cells for each treatment, presenting a 1.5-fold increase in γ-H2AX signal in the combined treated cells (Figure 4C; A549 left panel, H1975 right panel). Cell treated with c#2 or c#3 did not exhibit high foci number/cell or bright signal and were similar to DMSO treatment; therefore, they are not presented here. These results suggest that combined treatment indeed causes an increase in DNA damage, which correlates with enhanced cell death.

### 2.4. MAD2L2-Rev1 Interaction Is Reduced after Exposure to c#2 or c#3

**Figure 5:**
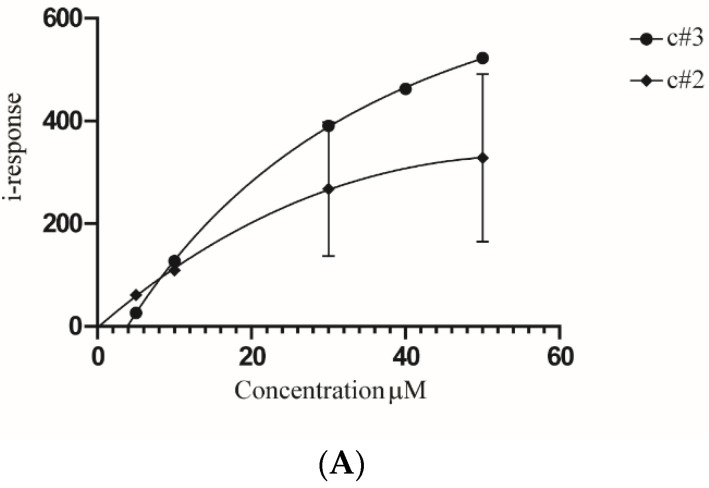

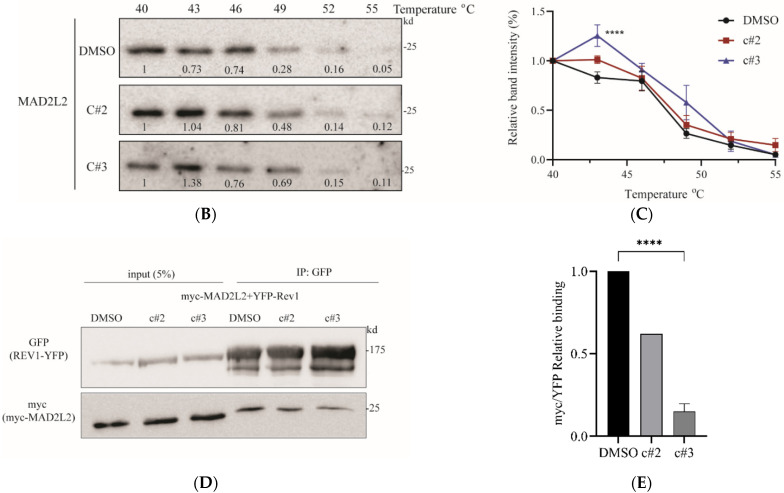
Compounds #2 and #3 reduce Rev1-MAD2L2 interaction. (**A**) Binding activity of the compounds c#2 and c#3 to MAD2L2 in vitro. Binding assay of increasing concentrations of compounds c#2 and c#3 to MAD2L2. c#2 k_D_ = 310.8 ± 32.96 μM; c#3 k_D_ = 44.88 ± 2.062 μM. (**B**) MAD2L2′s cellular thermal shift assay (CETSA) in 293T cells after 1-h treatment with DMSO or 50 μM of c#2 or c#3. Relative MAD2L2 levels at each temperature compared to DMSO are indicated. (**C**) Aggregation curve generated by quantification and comparison of MAD2L2 in each cellular thermal shift assay. *n* = three independent experiments. *p*-value < 0.0001 (****) was calculated for row factor of c#3 compared to DMSO using a two-way ANOVA. The additional blots contributing to this analysis are shown in Figure S2A. (**D**) HEK293 cells were co-transfected with YFP-Rev1 together with myc-MAD2L2, and an α-GFP IP was performed to assess Rev1-MAD2L2 binding when treated with 50 μM of each compound. (**E**) Quantification of the relative amount of myc-MAD2L2 bound to YFP-Rev1. For DMSO and c#3 *n* = 3 independent experiments, for c#2 *n* = 1 independent experiment, SD = 1, error bars represent 1 SD. *p*-value< 0.0001 (****) was calculated by two-tailed *t*-test. The additional co-IP blots contributing to this analysis are shown in Figure S2B.

In order to validate that MAD2L2 is a direct target of c#2 and c#3, we tested the compounds’ binding kinetics to a recombinant protein by field-effect biosensing (FEB) technology. Briefly, purified GST-MAD2L2 was immobilized onto a graphite chip, and the current across the chip was measured to determine the baseline current. Then, a soluble compound flowed over the chip. Interaction of the soluble and immobilized molecules yields a detectable change in conductance of the biosensor. The k_on_ and k_off_ of the interaction were measured over time and were used to calculate the k_D_. The binding of both c#2 and c#3 to MAD2L2 is in the micromolar range: 310.8 μM and 44.88 μM, respectively (Figure 5A). The affinity of c#3 to MAD2L2 is approximately seven-fold higher in comparison to the affinity to c#2. Next, the in vivo binding of c#2 or c#3 to MAD2L2 was validated using a cellular thermal shift assay (CETSA), which is a reliable reporter showing whether a molecule enters the cell and the nucleus and binds to the protein of interest [20]. Briefly, under normal conditions, most of the MAD2L2 protein unfolds, aggregates and disappears from the soluble fraction of the lysate between 46 °C and 49 °C. A change in the aggregation temperature is expected upon direct binding of a compound, as the compound will affect the relative stability of the folded protein and either promote or delay its unfolding and subsequent aggregation. In this assay, we show that both c#2 and c#3 stabilize the folded MAD2L2 protein through direct binding, raising the aggregation temperature. Figure 5C presents the temperature-induced aggregation generated after 1 h of compound treatment and exposure of cells to a temperature gradient of 40–55 °C. Under normal conditions, MAD2L2 aggregates in the shift between 46 °C and 49 °C. However, when c#3 is added, MAD2L2 remains soluble until higher temperatures, and the aggregation occurs between 49 °C and 52 °C (Figure 5B,C). These data suggest that c#3 binds MAD2L2 and changes its solubility. C#2 caused a milder change in MAD2L2′s aggregation temperature, suggesting a weaker binding to MAD2L2.

In order to determine whether both compounds interfere with MAD2L2-Rev1 complex formation in vivo, we performed co-immunoprecipitation (co-IP) of the complex. HEK293 cells were co-transfected with YFP-Rev1 together with myc-MAD2L2. Transfected cells were treated with either DMSO or with the compounds for 24 h before the MAD2L2-Rev1 complex was IP’ed using α-GFP-Trap beads, which recognize YFP. While YFP-Rev1 presented a clear binding to myc-MAD2L2 when no compound was added, a clear reduction was observed in MAD2L2-Rev1 binding after 24 h exposure to c#2 or c#3 (Figure 5D). A difference between c#2 and c#3 was observed: while c#2 did not reduce complex formation significantly, c#3 was extremely effective and reduced MAD2L2-Rev1 binding by close to 80% (Figure 5E). The biochemical data support the idea that c#3 binds better to MAD2L2 than c#2, and c#3 is probably more efficient in disrupting the MAD2L2-Rev1 complex in vivo. Hence these compounds’ biological effect is due to their direct binding to MAD2L2.

## 3. Discussion

In this study, we discovered two small molecules that potentially disrupt the assembly of MAD2L2-Rev1 and the formation of an active TLS complex. Using molecular dynamics simulation, we generated a new model of the MAD2L2 homodimer together with one Rev1 protein. This model exposed a unique cavity formed upon MAD2L2 homodimerization, which could serve as a new binding interface for small molecules. Hence, the MAD2L2 homodimer model was applied to a docking simulation to identify small molecules that potentially bind in this unique cavity. We performed an unbiased bioinformatics screen of approximately 5 million molecules that are available in the ZINC12 subset library. Two small molecules, c#2 and c#3, were selected for further analysis. Both compounds have lead structures, MW ≤ 350 and the predicted octanol-water partition coefficient XlogP [23] ≤3.5. Thus, both serve as a good starting point of lead optimization in order to improve their potency, selectivity, and pharmacokinetic parameters. Hence, our approach demonstrates the importance of high-resolution structures of protein complexes that can be used for in silico drug design.

First, we observed that both c#2 and c#3 sensitized lung cancer cell lines to cisplatin, resulting in significant cell death. In H1975 cells, a clear reduction in the measured IC50 of cisplatin and doxorubicin was observed. However, A549 cells were less sensitive to the compounds, as IC50 was reduced by about one-third after combined treatment, while in the H1975 cells, combined treatment reduced IC50 by half (Figure 3). The source of the sensitivity difference between cell lines is not determined; however, it could be related to different expressions of multidrug resistance (MDR), or differences in basal TLS activity and other DNA damage complexes.

MAD2L2 is important for DNA damage tolerance and repair. Therefore, we hypothesized that the hypersensitization of the cells to cisplatin in the presence of c#2 or c#3 is the result of persistent cisplatin-induced DNA damage. Indeed, both molecules caused an increase in DNA damage only when combined with cisplatin. Moreover, combined treatment caused a significant increase in the number of cells with more than 80 γH2AX foci/cell and extremely high signal, indicating that these cells suffer from severe levels of DNA damage (Figure 4C,D). Importantly, most of the cells with high γH2AX presented round and intact nuclei, suggesting that they suffer from DNA damage, but they are not fully apoptotic cells. Notably, c#3 and c#2 alone did not cause any increase in γH2AX signal, and cell nuclei remain intact. Interestingly, this effect was more prominent in c#3 than c#2, as c#2 increased the highly damaged cells only in A549 cells. This might be due to the higher cisplatin concentration used in the assay for A549 cells (10 μM instead of 5 μM cisplatin), as A549 cells presented intrinsic resistance to the combined treatment.

The binding of c#2 and c#3 to MAD2L2 has been confirmed in vitro. We determined the k_D_ of the compounds to be in the micromolar range (Figure 5A). Interestingly, the affinity of c#3 to MAD2L2 is sevenfold higher than c#2. Furthermore, the R squared value of c#2 is lower than c#3, suggesting that its binding to MAD2L2 is less stable. Next, we performed in vivo CETSA to assess the binding affinity of c#2 and c#3 in cells (Figure 5B,C). In agreement with our in vitro measurements, c#3 presented a stronger binding affinity to MAD2L2 than c#2. Finally, we explored whether the biochemical interaction affects the MAD2L2-Rev1 binding. Both molecules interfere with MAD2L2-Rev1 binding; however, as expected from the measured affinities, c#3 is more potent than c#2 in preventing complex formation (Figure 5D,E). Interestingly, despite the superior binding of c#3 to MAD2L2, no significant difference was observed between the molecules in cell sensitization. One potential explanation might be that the weak binding of c#2 to MAD2L2 may reduce the number of active TLS complexes, slowing TLS activity and potentially slowing the turnover of TLS proteins. This might explain the reduction of highly damaged cells when exposed to low cisplatin concentration, as TLS might still be functioning and preventing their appearance. The c#3 molecule efficiently prevents MAD2L2-Rev1 complex formation and may inhibit TLS activity better than c#2, causing accumulation of highly damaged cells, even at a low cisplatin concentration. More research is still needed to gain a better understanding regarding the nature of the interaction of these molecules with MAD2L2.

Finally, in addition to its role in TLS, MAD2L2 has central roles in different cellular pathways [24]. Recently, it was found to be part of the shieldin complex, promoting non-homologous end-joining and inhibiting homologous recombination (HR) [25,26,27]. In the shieldin complex, MAD2L2 homodimerization and the activity of TRIP13 were found to be crucial for its proper regulation [22,28,29,30]. Moreover, recent evidence links mutations in the shieldin complex to resistance to PARP inhibitors in BRCA 1/2-deficient cells [30,31]. Moreover, MAD2L2 overexpression was found in several cancer types and is correlated with poor prognosis [24,32,33].

In addition, MAD2L2 was found to be involved in several aspects of mitotic regulation. MAD2L2 inhibits the anaphase-promoting complex/cyclosome (APC/C) [34,35,36] and has been shown to bind RAN GTPase and the chromosomal alignment protein CAMP [20,37,38], helping to stabilize the mitotic spindle. However, the influence of both compounds on other MAD2L2-related pathways is yet to be evaluated.

Thus far, inhibition of TLS has been done using genetic methods, strengthening the concept that TLS inhibition is a promising target for cancer treatment. Inhibiting Rev1 using JH-RE-06 or Rev3 presented promising results for cancer treatment [16,25,39]. However, the beneficial effect of these inhibitors is still under debate [40]. Considering these recent results, identifying and developing additional TLS inhibitors is proving to be crucial in the ongoing effort of improving cancer patient therapy and prognosis.

## 4. Materials and Methods

### 4.1. Generating MAD2L2-MAD2L2-Rev1 Model

The dimeric form of the structure of MAD2L2 in complex with a CAMP fragment was built from the monomeric structure (PDB code 5XPT [21]) using the crystal symmetry matrix as shown in the above paper. Rev1 was added to one of the MAD2L2 monomers by the superimposition of the MAD2L2-Rev1 complex structure (PDB 3VU7 [41]). The position of Rev1 was further refined by performing local docking of Rev1 to the MAD2L2 dimer using the Rosetta Online Server [42,43,44]. The top five docking results were examined, and three of them were similar. The middle pose of the three similar results was selected for molecular dynamics simulation, and missing amino acids in the structure were added using Modeller [45]. Molecular dynamics simulations were carried out using GROMACS software using the AMBER36 force-field and TIP3P water model with an ionic strength of 100 mmol/L NaCl. The system was backbone-restrained and energy-minimized. This was followed by a two-step equilibration process of 100 ps to the target temperature and pressure of 310 K and 1 bar. Backbone restraints were lifted, and a 30 ns-long full MD simulation with a 2-fs time step under constant pressure and temperature was conducted. Snapshots were taken every 0.1 ns. The last 10 frames were compared by RMSD, and the conformation that showed the highest similarity to the other frames was selected for docking simulations.

### 4.2. Docking Simulations

High-throughput virtual screening virtual docking of small molecules was carried out in the cavity of the MAD2L2 homodimer interface of the above conformation after removing Rev1. Docking simulations were done using the University of California at San Francisco, San Francisco, CA, USA (UCSF Dock; v6.8 with ZINC12 Clean Leads subset of small ligands (Subset ID 11) [46,47,48]. This dataset contains 4,591,276 molecules selected according to the following criteria: p.mwt ≤ 350 and p.mwt ≥ 250 and p.xlogp ≤ 3.5 and p.rb ≤ 7. First, all molecules were docked using the flex anchor-and-grow (fast) method for a rigid receptor and flexible ligand with grid-based scoring. After an initial large-scale scanning, the best 5000 compounds were docked and rescored using AMBER force files, with default scoring parameters allowing flexibility for the ligand and receptor amino acids with a cut-off distance of 2.5 Å from the ligand. Molecules making less than 2 Hb with the MAD2L2 homodimer or having an Amber_Score binding energy of less than −10 were filtered out. The final selection step was done via manual selection, in which the remaining candidate ligand-protein complexes were visually scanned and ranked from 1 (compound binding at residues not essential for MAD2L2-Rev1 binding) to 4 (compound binding tightly at residues essential for MAD2L2-Rev1 binding). Specifically, the compounds predicted by visual estimation to bind most closely to the K1203 and L1205 interface residues of Rev1 (essential MAD2L2 binding residues) were ranked highest (4). Of the compounds ranked as 4, the 10 compounds available for purchase from suppliers were selected for experimental validation.

### 4.3. Protein Induction, Purification and Field Effect Biosensing (FEB)

*E. coli* BL21 Star cells were transfected with GST-MAD2L2 vector. Protein induction was done for 4 h at 30 °C with 1 mM IPTG. Purification was done according to standard GST protocol.

The binding, kinetics of MAD2L2 to the small molecule compounds were measured by the field-effect biosensing (FEB) Agile R100 label-free binding assay (Nanomedical Diagnostics Inc, San Diego, CA, USA), following their standard protocol [49,50]. Briefly, 500 nM of purified MAD2L2 was immobilized on a graphene sensor chip through amine groups. The current baseline level for the chip was recorded in PBS. Next, PBS was aspirated, and the changes in the baseline current induced by 50 µL of 1, 10, 20, 30, 40 and 50 μM droplets of the tested compound were recorded. k_D_ values were calculated by the DataLine 2.0 software by either a Hill equation fit or by using the k_on_ and k_off_ values at a single concentration. The k_D_ values obtained by these two methods were almost identical.

### 4.4. Cell Culture, Plasmids and Transfections

The A549, HEK293, and 293T cells were cultured in Dulbecco’s modified Eagle medium/DMEM (Biological Industries, Kibbutz Beit-Haemek, Israel; 01-052-1A) with 4.5 g/L D-glucose, 4 mM L-glutamine, 10% fetal bovine serum (Biological Industries Israel Beit Haemek; 04-007-1A), and 1% penicillin/streptomycin. The H1975 cells were cultured in RPMI 1640 medium (Biological Industries, Kibbutz Beit-Haemek, Israel; 01-100-1A) with 4.5 g/L D-glucose, 4 mM L-glutamine, 10% fetal bovine serum (Biological Industries, Kibbutz Beit-Haemek, Israel; 04-007-1A), and 1% penicillin/streptomycin. MAD2L2-myc was constructed in pcDNA3.1(+) (Life Technologies) by fusion of a myc tag to the C terminus of full-length human MAD2L2. REV1-YFP plasmid was a gift from Prof. Zvi Livneh, Weizmann institute, Rehovot, Israel. Transfections of HEK293 cells with plasmid DNA were performed using the Avalanche Everyday Transfection Reagent (EZT-EVDY-1) according to the manufacturer’s protocol. Briefly, cells were passaged the day before transfection to reach a confluency of 60–70%. The next day, the selected plasmid DNA was incubated in serum-free media with the recommended volume of transfection reagent for 20 min at room temperature. This transfection mix was gently added to the prepared cell culture plate(s) for continued incubation at 37 °C. For co-IP experiment plates, after 24 h, the relevant compound (DMSO vehicle, c#2, or c#3) was added in pre-warmed media, and the transfected plates were incubated for another 24 h at 37 °C until harvesting.

### 4.5. Colony Survival Assay

Cells were plated in 24-well plates at 50,000 cells/well (H1975 line) or 10,000 cells/well (A549 line). The next day, cells were treated with varying concentrations of cisplatin (0–10 μM), with or without 50 μM compound. For the untreated control wells containing only cisplatin, DMSO was applied in place of the compound. After 48 h of cisplatin + compound treatment, the cells were washed 3 times with PBS, and fresh media was applied. After 4 days of recovery, the cells were stained with methylene blue and imaged using a Nikon SMZ25 stereomicroscope. The images were analyzed using the ImageJ (National Institutes of Health, Bethesda, MA, USA) area measurement tool to quantify the total colony size in each well.

### 4.6. Western Blot and Co-Immunoprecipitation

HEK293 cells were harvested 48 h following co-transfection and lysed in extraction buffer (50 mM Tris pH 8, 150 mM NaCl, 20 mM EDTA, 50 mM NaF, 1% TritonX) supplemented with Merck 1000× protease inhibitor (539,134). Cells were lysed on ice for 30 min and centrifuged at 20,000× *g* for 30 min at 4 °C. For immunoblotting, extracts were boiled in Laemmli buffer for 5 min. Equal amounts of protein sample (30 µg) were loaded on 8–12% acrylamide gel and transferred to a nitrocellulose membrane (Amersham). For immunoprecipitations, clarified lysates were supplemented with 7 µL of equilibrated GFP-Trap antibody-conjugated agarose beads (Chromotek; gta-20, Munich, Germany) and incubated for 1–2 h at 4 °C. Beads were washed 3 times in PBS buffer and boiled in Laemmli buffer for 5 min. The following primary antibodies were used for immunoblotting: Myc (Santa Cruz; SC-40, Santa Cruz, CA, USA) 1:1000 dilution, GFP (Santa Cruz, SC-9996) 1:1000 dilution, MAD2L2 (ProteinTech; 12683-1-AP, Rosemont, IL, USA). Appropriate light-chain-specific secondary antibodies were used at 1:10,000 for all membranes: anti-mouse (Jackson ImmunoResearch Laboratories, Inc; 115-035-174, West Grove, PA, USA) and anti-rabbit (EMD Millipore; MAB201P, Burlington, MA, USA). The membranes were developed with the BioRad biomolecular imager, and band densitometry was performed using the ImageJ (National Institutes of Health, Bethesda, MA, USA) Gel Analyzer function. All statistical analysis was performed with Prism 9.1.2 (GraphPad Software, San Diego, CA, USA).

### 4.7. Immunofluorescence

H1975 and A549 cells were grown on glass coverslips in 12-well plates with the appropriate media and fixed in 4% paraformaldehyde for 10 min at room temperature. Cells were permeabilized in 0.5% Triton×100 in PBSx1 for 10 min at room temperature and then blocked in 5% BSA in 0.1% PBSx1-Tween for 1 h at room temperature. Anti-phospho-Histone H2A.X primary antibody (Mercury; 05-636-25UG) diluted 1:400 in 5% BSA in 0.1% PBSx1-Tween was added for 1 h at room temperature. Fluorescent-dye conjugated secondary antibody was applied for 1 h at room temperature. The coverslips were washed between each step with PBSx1. Nuclei were stained with DAPI (1:2000 dilution) at room temperature in the dark for 3 min. Coverslips were mounted on glass slides and imaged using an Olympus 1 × 81 microscope. For all treatments, cells with fewer than 80 foci were scored manually (Figure 4B), and cells with more than 80 foci and very bright signals were scored automatically by using the “Analyze Particles” function in ImageJ (National Institutes of Health) after setting a suitable threshold.

### 4.8. Cellular Thermal Shift Assay (CETSA)

The in vivo binding of MAD2L2 to the small molecule compounds was detected using the cellular thermal shift assay (CETSA) following their standard protocol [51,52]. Briefly, 293T cells were grown to confluency and treated for 1 h with the DMSO or selected compounds at 50 μM. Cells were collected and centrifuged for 3 min at 300 g, washed once with PBS, and each pellet was resuspended in 1ml of PBS supplemented with Merck 1000× protease inhibitor (539134). The cell suspensions were aliquoted and incubated for 3 min in the VWR XT96 thermal cycler using a gradient from 40–55 °C. Following heating, the tubes were incubated at room temperature for 3 min and then snap-frozen in liquid nitrogen. The samples were then lysed via 2 subsequent freeze-thaw cycles with vortexing, and the lysates were centrifuged at 20,000× *g* for 20 min at 4 °C. Finally, the cleared lysates were boiled in Laemmli buffer for 5 min.

## 5. Patents

Patents resulting from the work: USA Provisional 63/109,521.

## Data Availability

Not applicable.

## Supplementary Materials

The following supporting information can be downloaded. Figure S1: Compounds #2 and #3 sensitize cells to cisplatin treatment. (A) Compounds #2 and #3 present no toxicity. (B) Compound #2 sensitizes cells to doxorubicin treatment. (C) Representative colony survival assay. Figure S2: Compounds #2 and #3 reduce Rev1-MAD2L2 interaction. (A) Additional blots that have been included in the CETSA quantification in Figure 5C. (B) Additional co-IP blots that have been included in the quantification in Figure 5E.

## Sample Availability

Samples of the compounds are available from the authors.
