# Supplementary material for: Disrupting the MAD2L2-Rev1 Complex Enhances Cell Death upon DNA Damage
Source: Molecules. 2022 Jan 19;27(3):636. doi: 10.3390/molecules27030636 (PMC8838411; doi:10.3390/molecules27030636)
Supplement: Supplementary file 1 [file molecules-27-00636-s001.zip › molecules-1518767-supplementary.pdf]

Supplementary Figures

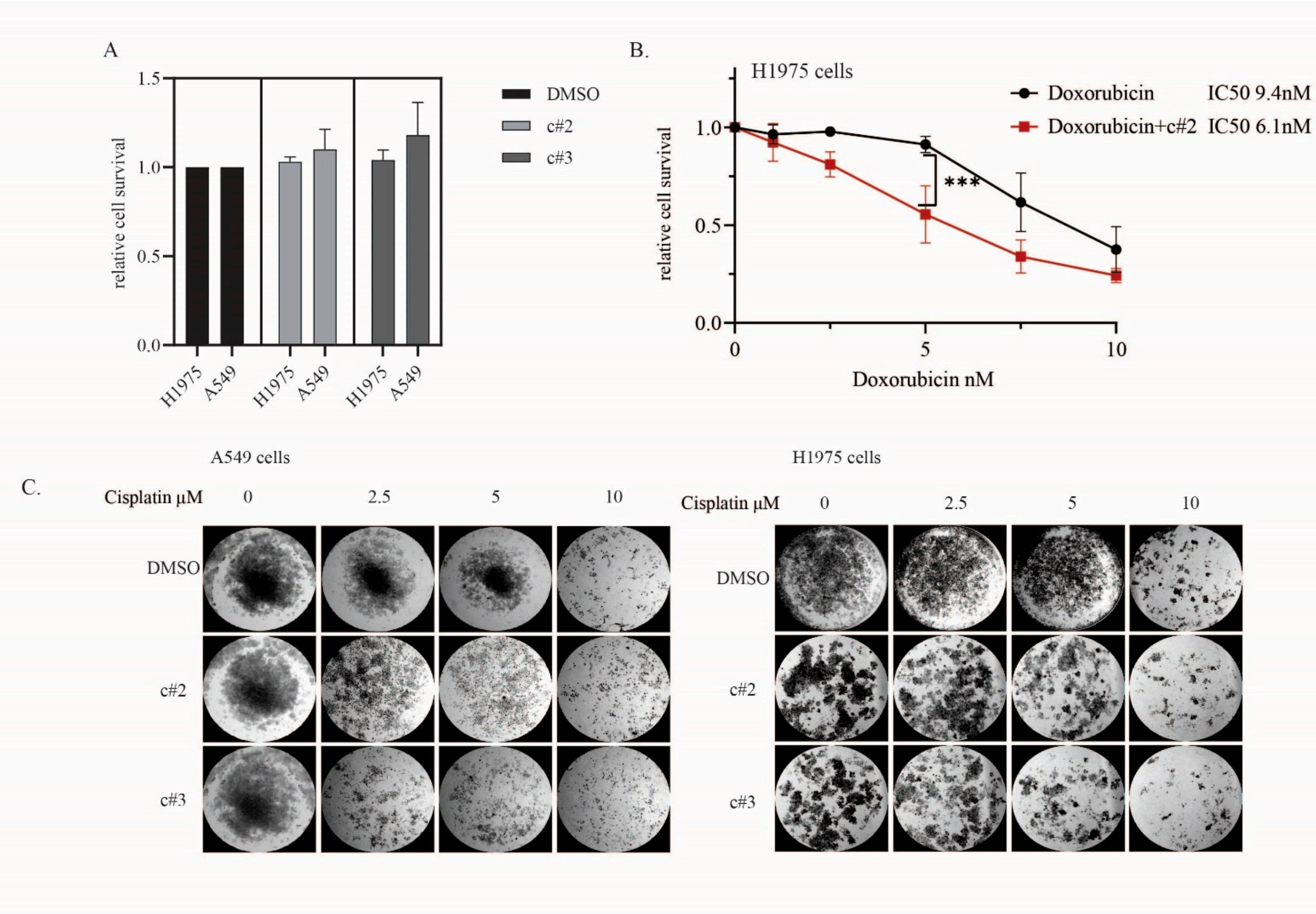

Figure S1. Compound c#2 and c#3 sensitizes cells to cisplatin. (A) Compounds #2 and #3 present no toxicity. H1975 and A549 cell lines presented no reduction in cell viability after performing the colony survival assay with 50 µM of each compound alone. (B) Compound #2 sensitizes cells to doxorubicin treatment. Colony survival assay of H1975 cell line. Cells were treated with 50 µM of c#2 and the indicated concentration of doxorubicin for 48h, then cells were washed in PBS and growth media was replaced with normal growth media with no compound. Cells were allowed to recover 4-5 days before staining. n=3 independent experiments, SD=1. P value was calculated by one tailed t-test. IC50 was calculated using linear regression. (C) Colony survival assay with c#2 and c#3 and increasing cisplatin concentrations. Representative figure of a colony survival assay with c#2 and c#3, that were used to generate the graphs in Figures 3A and 3B.

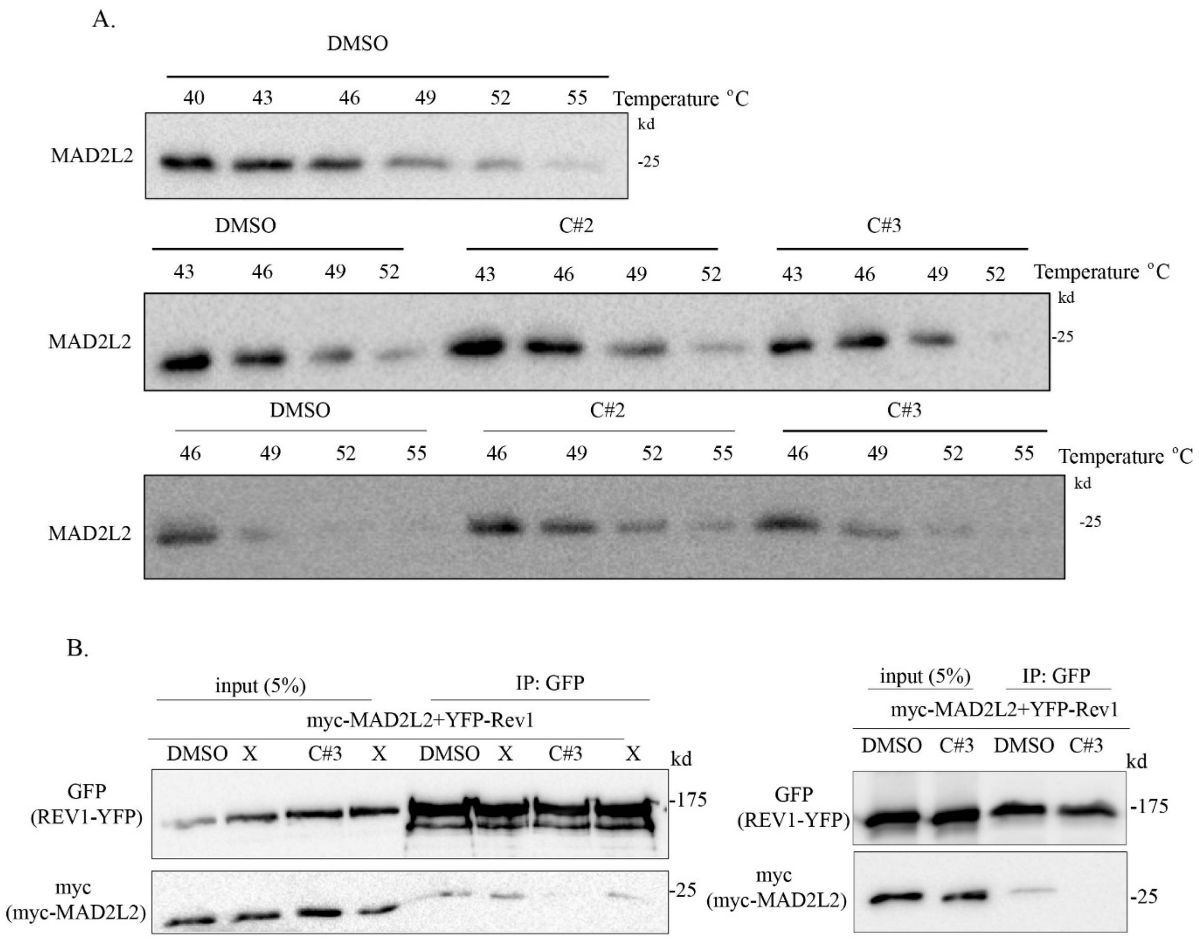

Figure S2. **Compounds #2 and #3 reduce Rev1-MAD2L2 interaction.** (A) Additional blots that have been included in the CETSA quantification in Fig. 5C. (B) Additional co-IP blots that have been included in the quantification in Fig. 5E.
